# Isolation and characterization of a novel exopolysaccharide from the fermented probiotic *Lactiplantibacillus plantarum* ZZU-1 and its application for attenuating autism-like behaviors

**DOI:** 10.3389/fmicb.2026.1806688

**Published:** 2026-07-08

**Authors:** Shuai Zhao, Mengyi Sun, Hanyan Li, Ziyi Wang, Ziliang Meng, Yuhan Xu, Siyi Mao, Wenchen Wang, Haowei Sun

**Affiliations:** 1 School of Biological Engineering, Henan University of Technology, Zhengzhou, China; 2 College of International Education, Henan University of Technology, Zhengzhou, Henan, China

**Keywords:** antioxidants, autism, exopolysaccharides, gut microbiota, *Lactiplantibacillus plantarum* ZZU-1

## Abstract

Lactic acid bacteria-derived exopolysaccharides (EPS) are natural and safe functional biomolecules whose antioxidant potential is largely dependent on their specific chemical structures. Accumulating evidence suggests that LAB-EPS may exert indirect regulatory effects on oxidative stress-related diseases like autism spectrum disorder via modulating intestinal microecology and relieving oxidative stress in the gut-brain axis. In this study, a novel EPS (EPS-ZZU) was isolated from *Lactiplantibacillus plantarum* ZZU-1 of traditional fermented Suancai. Structural characterization revealed a 2.141 kDa molecular weight, with mannose, glucose and ribose in a 34.40:26.35:12.24 molar ratio, composed of α-configuration pyranose units. EPS-ZZU exhibited over 90% scavenging rates against the typical free radicals, including hydroxyl radical (⋅OH), 1,1-diphenyl-2-picrylhydrazyl radical (DPPH•), superoxide anion (O_2_^•⁣–^) and 2,2’-azinobis (3-ethylbenzothiazoline-6-sulfonate) cation radical (ABTS•^+^) at a concentration of 5 mg/mL, which was comparable to that of vitamin C (Vc). In a one-month mouse trial, EPS-ZZU significantly alleviated autism-like behaviors (social deficits, repetitive actions) by reducing oxidative stress and inflammation, enhancing intestinal barrier integrity, and reshaping gut microbiota-enriching beneficial taxa (*Adlercreutzia, Christensenellaceae*) and inhibiting pathogens (*Erysipelatoclostridium*). Metabolomics confirmed upregulated indole-3-acetate and downregulated cognitive impairment-associated metabolites (asymmetric dimethylarginine, homogentisic acid). These findings highlight EPS-ZZU’s therapeutic potential for autism and provide a new idea for developing more bioactive bacterial EPS antioxidants.

## 1 Introduction

Autism spectrum disorder (ASD) is a neurodevelopmental disorder characterized by persistent deficits in the ability to initiate and sustain reciprocal social interaction and social communication (Le et al., 2024). Notably, no pharmacological interventions have been shown to effectively address the core symptoms of ASD (Qi et al., 2024). Importantly, ASD is often accompanied by a variety of comorbid conditions that further increase its clinical complexity and functional impairment, including attention-deficit/hyperactivity disorder (ADHD), anxiety and depressive disorders, intellectual disability, epilepsy, gastrointestinal (GI) disorders (e.g., constipation, diarrhea, and abdominal pain), and sleep disturbances (Lord et al., 2020). In recent years, growing evidence from multiple disciplines-including microbiology, neuroscience, and immunology-has implicated oxidative stress in the pathogenesis of ASD (Chen et al., 2024). Individuals with ASD frequently exhibit elevated oxidative stress, which is often associated with gut microbiota dysbiosis, a condition characterized by altered microbial composition and metabolic activity (Wan et al., 2024). Furthermore, an imbalanced gut microbiome may promote the excessive production of short-chain fatty acids (SCFAs), such as propionic acid. This SCFA can easily cross the blood-brain barrier, disrupt mitochondrial electron transport, and enhance reactive oxygen species (ROS) generation (Bhandari et al., 2020). This process forms a vicious cycle that aggravates oxidative stress and contributes to neuroinflammation and synaptic dysfunction (Kunst et al., 2023). Therefore, targeting the gut microbiota to mitigate oxidative stress represents a promising therapeutic approach for ASD.

Recently, several common approaches that regulating the gut microbiota to reduce oxidative stress have been proposed. Among them, probiotics intervention has gained more attention due to its high effective and safety (Shabbir et al., 2021). *Lactiplantibacillus plantarum* (*L. plantarum*), previously designated as *Lactobacillus plantarum*, is a highly versatile microorganism with an extensive ecological distribution, colonizing diverse niches ranging from the human gastrointestinal (GI) tract to various fermented foods (Echegaray et al., 2023). *L. plantarum* has holds the Qualified Presumption of Safety (QPS) designation issued by the European Food Safety Authority (EFSA) and has also obtained the “generally recognized as safe” (GRAS) certification from the U.S. Food and Drug Administration (FDA) (EFSA Panel on Biological Hazards (Biohaz), Ricci et al., 2017). It has been reported a significant constituent of probiotics, known for its many functional properties such as improves intestinal barrier integrity, modulates mucosal homeostasis, and exhibits antioxidant effects (Yang et al., 2025). Recent evidence has confirmed that *L. plantarum* exerts vital effects on alleviating autism spectrum disorder via multiple mechanisms. For instance, *L. plantarum* N-1 regulates gut microbiota and relieves autism-like behaviors in maternal immune activation-induced ASD mice (Qiu et al., 2023), while strain ST-III improves autistic symptoms by modulating intestinal flora composition (Zhang et al., 2022). Moreover, a randomized, double-blind, placebo-controlled trial using *L. plantarum* PS128 further demonstrated the beneficial effects of *L. plantarum* in the intervention of ASD (Liu et al., 2019). Despite the existence of certain studies that demonstrate connections between *L. plantarum* and the mitigation of ASD, the key material basis for the probiotics to improve ASD remains unclear.

Exopolysaccharides (EPS) are secondary metabolites of microorganisms, which can either be secreted into the extracellular environment or adhere to the surface of bacterial cells (Milosavljevic et al., 2022). It has garnered considerable attention in recent years due to various physiological effects antioxidant, anti-cancer, and immune-modulatory activities, as well as the capacity to promote probiotic colonization in the host intestinal tract (Ju et al., 2024). EPS produced by *Lactiplantibacillus* species are generally recognized as safe for human consumption (Prete et al., 2021) and have been exploited in diverse applications, including as potential natural, non-toxic food additives and therapeutic agents due to its functional properties especially antioxidant (Wei et al., 2024). EPS exhibit potent antioxidant properties through multiple synergistic mechanisms including scavenging reactive oxygen species (ROS), enhancing cellular antioxidant defense systems, and regulating gene expression (Gu et al., 2023). Several studies have demonstrated that EPS are supplemented in the treatment of human disorders such as cancer, atherosclerosis, rheumatoid arthritis, and neurodegenerative diseases by increased antioxidant activity (Angelin and Kavitha, 2020). These findings underscore the critical role of EPS in regulating redox homeostasis and mitigating oxidative damage. However, the precise effects and mechanism by which EPS alleviates ASD-related oxidative stress are still inadequately comprehended.

In this study, a novel EPS named EPS-ZZU exhibiting robust antioxidant capacity was purified from probiotic *L. plantarum* ZZU-1, which was isolated from traditional fermented foods “Suancai.” Then the EPS was characterized and investigated for its potential to act as a therapeutic agent to investigate its protective effects on abnormal behavior in rat model with valproic acid (VPA)-induced autism. Moreover, in order to underly potential mechanisms by which EPS reduce oxidative stress for ameliorating ASD symptoms microbial community and metabolome analysis were performed. The results would provide an idea for the development of more functional EPS antioxidants, and further offers a new potential therapeutic strategy for ASD.

## 2 Materials and methods

### 2.1 Reagents and chemicals

Valproic acid (VPA) (CAS: 1069-66-5; purity ≥98%) and Phosphate Buffered Saline (PBS) buffer were obtained from Macklin Biochemical Technology (Shanghai, China). Fermented “Suancai” samples collected from a local farmer in Northeast China, and De Man, Rogosa, and Sharpe (MRS) agar and broth from Difco (Detroit, MN, United States). The mouse malondialdehyde (MDA) ELISA kit was sourced from LSBio (Seattle, WA, United States). Assays for glutathione (GSH) and enzymatic activities (CAT, SOD, and GPx) were conducted using kits procured from Abcam (Cambridge, United Kingdom). The iQ™ SYBR^®^ Green Supermix was acquired from Bio-Rad (Hercules, CA, United States). All remaining chemical reagents were of analytical grade and purchased from Sigma-Aldrich (St. Louis, MO, United States). DNA oligonucleotides for PCR were synthesized by Sangon Biotech (Shanghai, China), and fluorescent nucleic acid stain GelGold was ordered from Biotium (Beijing, China).

### 2.2 Isolation, purification, and characterization of EPS

The EPS-ZZU from *L. plantarum* ZZU-1 was cultured in MRS medium and then extracted and purified according to the reported method with minor modifications (Wang et al., 2019), which the strain was isolated and identified from Suancai samples. To obtain EPS, the probiotic broth was filtered and centrifuged at 10,000 rpm with 10 min. The supernatant was concentrated to 1/5 of the original volume by a rotary evaporator, followed by adding 1/4 volume of Sevag solution (trichloromethane: 1-butanol/V: V = 4:1) for several times. The deproteinized supernatant was added to pre-chilled alcohol as four times of volumes and maintained at 4°C overnight. The precipitate was freeze-dried to obtain the crude EPS, which was then dissolved and dialyzed in distilled water with a molecular cutoff of 1,000 Da. The dialyzed solution was purified using a Sepharose CL-6B column (2.5 × 60 cm) with an elution phase of 0.1 mol/L NaCl at a flow rate of 0.6 mL/min after being filtered through 0.22 μm membrane. The final product was lyophilized to obtain the pure EPS. Characterization of the EPS was analyzed using scanning electron microscopic (SEM, FEI Quanta 250 FEG), atomic force microscope (AFM, NanoScopeIIIA controller, Bruker), fourier transform infrared spectroscopy (FT-IR, Bruker, Rheinstetten, Germany), nuclear magnetic resonance (NMR, Brucker, Rheinstetten, Germany) and ultraviolet-visible spectroscopy (UV-Vis, Implen Nanophotometer, Germany) as prior description (Lv et al., 2025). The molecular weight of EPS was determined by Agilent PL-GPC50 with a PLgel 8 μm aquagel-OH Mixed-M (7.5 × 300 mm) column (Agilent, United States) at 40°C and the flow rate was 1.0 mL/min (Liu et al., 2022) as well as a thermal analysis instrument (PerkinElmer STA6000, Perkin Elmer, United States) was used to test the thermal properties of the EPS (Huang-Lin et al., 2022). For monosaccharide compositions and protein detection, high performance liquid chromatography (HPLC, LC-20AD, Shimadzu, Japan) test based on prior study was performed (Kook et al., 2019) and the protein content of EPS was examined according to the Coomassie Brilliant Blue method (Blumenkrantz and Asboe-Hansen, 1973).

### 2.3 Antioxidant capacity of the EPS-ZZU

The antioxidant activity of the EPS-ZZU along with vitamin C (Vc) concentration from 0 to 5 mg/mL was assessed using standardized protocols previously reported in the literature (Zhou et al., 2024). For hydroxyl radical (⋅OH) scavenging assay, the EPS-ZZU were mixed equally in volume to ferrous sulfate (6 mmol/L) and hydrogen peroxide and kept at room temperature for 10 minutes. After colour rendering with salicylic acid (6 mmol/L) for 30 minutes, the absorbance at 510 nm of the solution was measured (Hu et al., 2019). For DPPH (DPPH•) scavenging assay, 2 mL of 0.1 mmol/L DPPH dissolved in ethanol was added to EPS solution for 30 min. The absorbance values of the mixture were measured at 517 nm (Tang et al., 2017). The superoxide anion (O_2_^•⁣–^) scavenging abilities of EPS was measured according to the method described by Qiu et al. (2023) with little modification. The superoxide radical was generated in 4 mL of sodium phosphate buffer (0.2 M, pH 7.3) containing 20 μM phenazine methosulfate, 160 μM NADH, 55 μM NBT and EPS-ZZU samples. The mixture was incubated at room temperature for 10 min, and the absorbance of the mixture was measured at 560 nm (Li et al., 2014). ABTS radical (ABTS•^+^) scavenging activity was assessed using a standard colorimetric method as commercial assay kits (Nanjing Jiancheng Biological Technology Co., Ltd., Nanjing, China) reported. ABTS^+^ working solution was prepared and diluted to an absorbance of 0.70 ± 0.02 at 734 nm. After mixing with the sample, the reaction system was incubated in the dark for 6 min, and the absorbance was determined at 734 nm. The free radical scavenging rate was calculated and expressed as percentage inhibition. All absorbance measurements were performed using a microplate reader (PerkinElmer, Germany) calibrated to ensure accuracy in quantification (Zhou et al., 2022).

### 2.4 Animal experiment protocol

**FIGURE 1:**
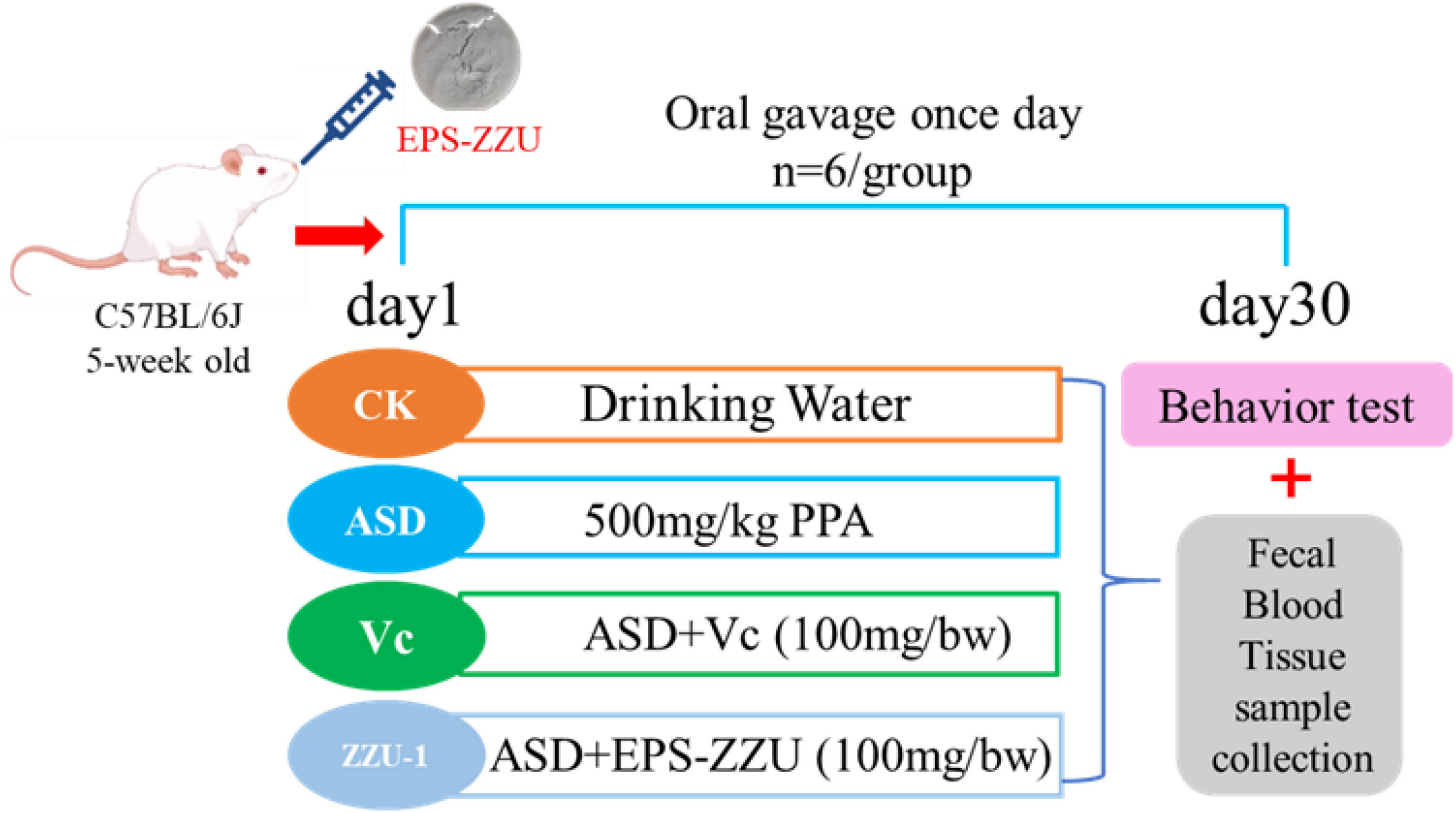
Flow diagram of the animal experiment design.

The study involved 24 healthy male Sprague-Dawley (SD) rats (body weight 150 ± 50 g) was procured from the Experimental Animal Center of Zhengzhou University. And the experimental was approved by the Medical and Experimental Animal Ethics Committee of Zhengzhou University (No: ZZU-LAC20221121[06]). The rats were housed adhering to the guidelines for laboratory animal care in a controlled environment related to 20°C–22°C, 50%–60% relative humidity with a 12-h light/dark cycle. Then they were randomly and evenly divided into four groups: control (CK), ASD model, ASD with Vc, and ASD with EPS-ZZU (Figure 1). For ASD induction, non-control groups received subcutaneous injections of valproic acid (VPA) at 500 mg/kg/day for five consecutive days, following a previously established protocol (Zhao et al., 2019). CK group was administered normal saline. Intervention groups received oral administration of EPS-ZZU or vitamin C with 100 mg/kg body weight (Barba-Vila et al., 2025). Post-treatment behavioral analysis was conducted after one month of intervention. Weekly monitoring included body weight, fecal consistency, and bleeding. Tribromoethanol was used as a general anesthetic agent with dosage of 150 mg/kg intraperitoneally according to the previous study (Gopalan et al., 2005). Blood was treated by posterior orbital plexus and centrifuged at 3,000 × *g* for 10 min to obtain serum sample. Hippocampal and Distal colon tissues (∼1 cm) were excised and fixed in 4% paraformaldehyde after euthanasia.

### 2.5 Behavioral assessments

The behavioral assessments were conducted by a blind experiment to group assignments to ensure objectivity. All experiments were performed between 09:00 and 17:00 under controlled lighting conditions (30–100 lux) to minimize circadian variations. In the open field test (OFT), rats were individually housed in a black plastic box (100 cm × 100 cm × 40 cm) divided into central and peripheral zones, with the central area defined as a 40 cm × 40 cm square. An overhead camera system coupled with Smart3.0 software (PanLab, United States) recorded their spontaneous locomotor activity by measuring the frequency of entries and duration spent in the central zone (Kraeuter et al., 2019). The three-chamber social test (TCT) was assessed using a transparent acrylic chamber (100 cm × 100 cm × 40 cm) connected to a three-chamber apparatus. After 10 min observed, with residence time in each chamber (S1, S2, and empty cage E1) measured using Smart3.0 software (Buffington et al., 2016). Additionally, the elevated plus maze test (EPM) was conducted according to established protocols to assess anxiety-related behaviors, including the time spent in open versus closed arms and the number of entries into these regions. The apparatus typically consists of two opposite open arms (50 cm × 30 cm) and two closed arms (50 cm × 30 cm), elevated 50 cm above the ground, with a central platform connecting all four arms (Zhang Y. et al., 2025). Novel object recognition (NOR) test was operated over a 3-day protocol, with the initial acclimation phase designed to minimize environmental stress (Govindaraj et al., 2017). On the first day, rats were habituated to the testing arena (60 × 60 × 60 cm^3^) for 5 min, ensuring they became familiar with the spatial context without object exposure 4. In the subsequent familiarization phase (Day 1), the old object [O] and familiar object [F] were positioned approximately 10 cm from the enclosure walls, with their spatial placement maintained consistently across all trials. Rats were allowed to engage in free exploration for 5 min, followed by thorough cleaning of the apparatus with 75% ethanol to eliminate olfactory cues. After 24 h, one familiar object was randomly exchanged for a novel object (N) of similar size but distinct shape and color, with object positions unchanged. Rats were reintroduced to the test box for a further five minutes of exploration prior to removal.

### 2.6 Biochemical analysis

The Hippocampal tissue samples of reduced glutathione (GSH), total superoxide dismutase (T-SOD), malondialdehyde (MDA), aspartate transaminase (AST), alanine transaminase (ALT), and blood urea nitrogen (BUN) were quantified using commercially available biochemical assay kits from Nanjing Jiancheng Bioengineering Institute (Nanjing, China). For the assessment of cytokine levels, including interleukin-6 (IL-6), interleukin-10 (IL-10), and tumor necrosis factor-alpha (TNF-α), rat-specific ELISA kits were employed according to the manufacturer’s protocol (Fankel, Shanghai, China). Intestinal permeability was evaluated by measuring serum levels of D-lactate (D-Lac) and diamine oxidase (DAO). Blood samples were collected under aseptic conditions and allowed to clot at room temperature for 30 min, followed by centrifugation at 3,000 rpm for 15 min at 4°C. The supernatant (serum) was carefully harvested, aliquoted into sterile centrifuge tubes, and stored at −80°C until analysis. Serum D-Lac concentration and DAO activity were determined using commercial assay kits (Nanjing Jiancheng Biological Technology Co., Ltd., Nanjing, China) strictly in accordance with the manufacturer’s instructions. Briefly, standard solutions and serum samples were added in duplicate to a 96-well plate, followed by the addition of corresponding reagents and incubation at 37°C for the recommended duration. The absorbance (optical density, OD) was measured at 450 nm using a microplate reader. The concentrations of D-Lac and DAO activity were calculated based on the standard curve constructed simultaneously with each assay.

### 2.7 Hematoxylin and eosin staining and immunohistochemistry

Hippocampal tissues were processed following the previous protocols (Mebratie and Dagnaw, 2024). Tissues were initially fixed in 4% paraformaldehyde (PFA) in phosphate-buffered saline (PBS) at 4°C for 24 h, followed by dehydration via graded ethanol and embedding in paraffin wax. Serial sections of 5 μm thickness were cut using a microtome and mounted on charged slides. Standard hematoxylin and eosin (H&E) staining was performed for histological evaluation. Immunohistochemistry (IHC) was conducted on paraffin-embedded sections. Sections were dewaxed, rehydrated, and subjected to antigen retrieval using 10 mM citrate buffer (pH 6.0) for 20 min at 100°C. Endogenous peroxidase activity was blocked with 3% H_2_O_2_/methanol for 15 min. Blocking was performed with 5% normal goat sample in PBS for 1 h at room temperature. Primary anti-Iba1 antibody (1:2,000; Cell Signaling Technology) was applied overnight at 4°C. After five washes with PBS, the secondary antibody (HRP-labeled rabbit anti-IgG; Servicebio, China) was incubated for 1 h at room temperature in the dark. Color development was performed using 3,3’-diaminobenzidine (DAB) tetrahydrochloride, followed by counterstaining with hematoxylin. Following dehydration, sections were mounted in DPX. Images were captured using a Nikon imaging system (TS2) and quantified with ImageJ software (version 1.8.0.112).

### 2.8 Immunofluorescence analysis

Immunofluorescence analysis was performed on dewaxed coronal sections following antigen retrieval using a microwave-induced heating method with sodium citrate buffer (pH 6.0) for 20 min (Im et al., 2019). The primary antibody, rabbit anti-rat Iba1 (Cell Signaling Technology, 17198), was diluted at a 1:500 ratio in PBS and incubated overnight at 4°C in a light-shielded humidified chamber. Subsequently, the sections were washed three times with PBS and incubated with a secondary antibody, anti-rabbit IgG conjugated with Alexa Fluor 488 (1:1000 dilution in 1% BSA/PBS), for 1 h at room temperature. Nuclei were counterstained with DAPI (1:1000 dilution) for 10 min in the dark, followed by three rinses with PBS. Fluorescence imaging was captured using a confocal laser scanning microscope (Leica DMI8 DFC7000T) equipped with appropriate filter sets for excitation/emission wavelengths of 495/519 nm (green) and 578/603 nm (red). Image acquisition parameters were optimized to ensure consistent signal intensity and resolution across all samples.

### 2.9 Microbial community sequencing

Approximately 1.0 g of mid-stream fecal samples were collected and subjected to DNA extraction using a Fecal DNA Extraction Kit (cat. no. DP328-02; Tiangen Biochemical Technology Co., Ltd., Beijing, China). The V3–V4 hypervariable regions of the 16S rRNA gene were amplified with the primers 341F (5′-CCTACGGGNGGCWGCAG-3′) and 785R (5′-GACTACHVGGGTATCTAATCC-3′). After amplification, the PCR products were purified and subjected to agarose gel electrophoresis for amplicon separation. The target DNA fragments were excised from the gel, purified, and prepared for sequencing. Sequences sharing ≥97% similarity were clustered into operational taxonomic units (OTUs). The representative sequences of each OTU were used for taxonomic annotation and subsequent classification analyses.

### 2.10 Metabolomic analysis

A 50 mg fecal sample was accurately weighed into a 2 mL centrifuge tube, followed by addition of 600 μL methanol containing 2-chloro-L-phenylalanine. The mixture was vortexed for 30 s to homogenize thoroughly before 100 mg glass beads were added. Samples were ground at 60 Hz for 90 s, then ultrasonicated at room temperature for 10 min to enhance extraction. After centrifugation at 12,000 rpm for 10 min at 4°C, supernatants were filtered through 0.22 μm membranes to obtain filtrates. Untargeted metabolomics analysis was conducted using a Waters Acquity I-Class PLUS UHPLC system coupled with a Xevo G2-XS QT high-resolution mass spectrometer. Raw data acquired via MassLynx were processed using R package XCMS (v3.12.0). Metabolites exhibiting a relative standard deviation (RSD) > 30% in quality control (QC) samples were excluded. Identified compounds were annotated against the Human Metabolome Database (HMDB), MassBank, Kyoto Encyclopedia of Genes and Genomes (KEGG), LipidMaps, mzCloud, and the database from Panomix Biomedical Tech Co., Ltd. (Suzhou, China) to obtain classification and pathway information.

### 2.11 Statistical analysis

The data were expressed as mean ± standard deviation (SD) for continuous variables. For intergroup comparisons, normally distributed data were analyzed using the independent samples *t*-test, whereas non-normally distributed data were assessed via the Mann-Whitney U test. Categorical variables were evaluated using either the chi-square test or Fisher’s exact test, with the selection based on sample size and distributional characteristics. In cases involving multiple groups, the Kruskal-Wallis H test was applied for non-parametric data, whereas one-way ANOVA with *post hoc* Tukey pairwise comparisons was used for parametric data. All statistical analyses were conducted using GraphPad Prism (version 9.5.0). All *P*-values were two-tailed, with *P* < 0.05 considered statistically significant. The significance levels were denoted as follows: *P < 0.05, **P < 0.01, ***P < 0.001, ****P < 0.0001, ns, not significant.

## 3 Results and discussion

### 3.1 Probiotic ZZU-1 can produce EPS-ZZU

**FIGURE 2:**
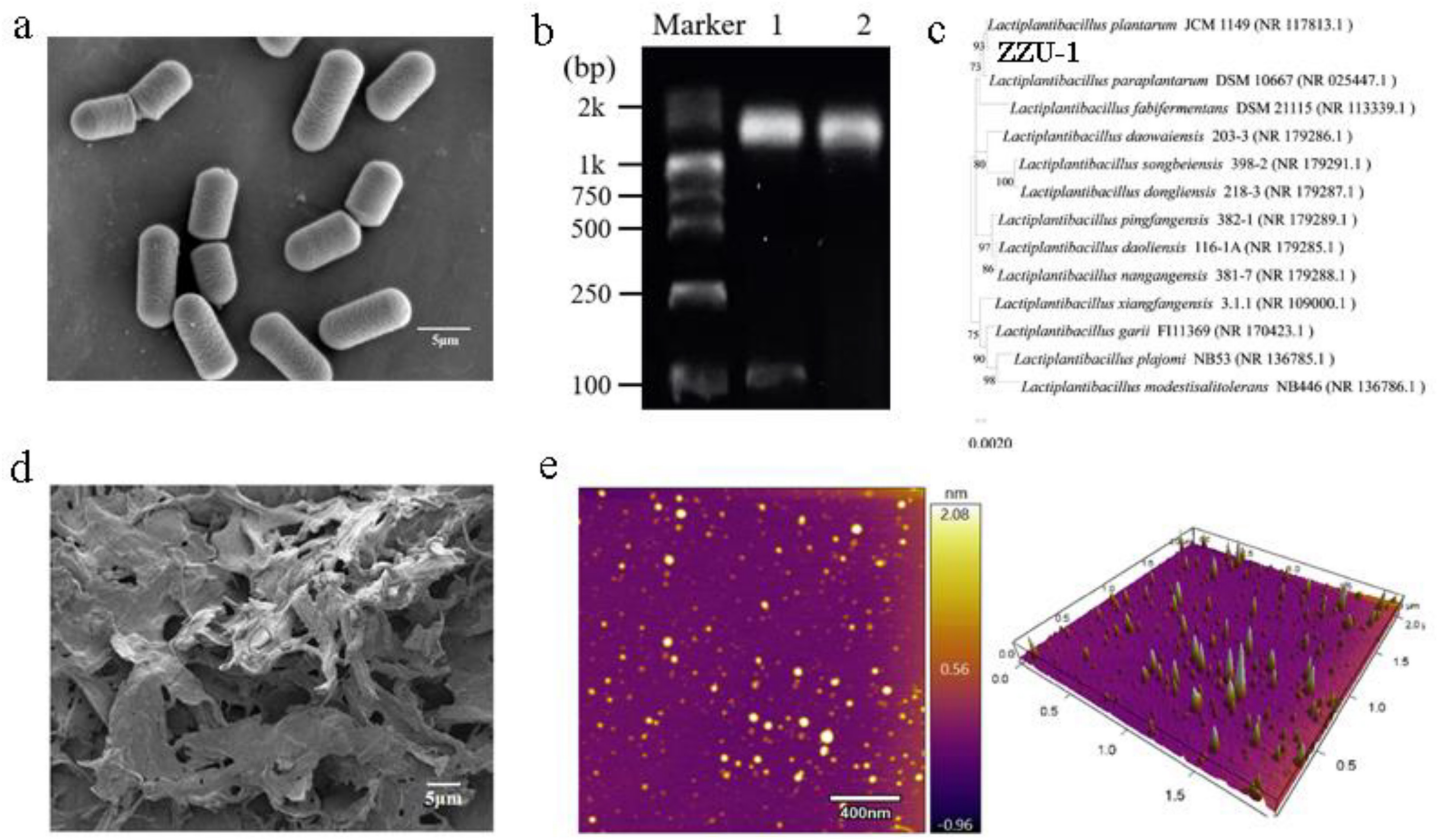
SEM images of the strain ZZU-1 **(a)**; Agarose gel electrophoresis of 16S rRNA from the strain ZZU-1 **(b)**; Phylogenetic tree of the strain ZZU-1 **(c)**; SEM **(d)**; and atomic force microscopy images **(e)** of the EPS-ZZU.

The strain ZZU-1 was isolated from fermented food Suancai. SEM analysis revealed that ZZU-1 possessed a rod-shaped and short morphology, which is consistent with the typical morphology of *Lactiplantibacillus* species (Figure 2a). To further identify the strain, the 16S rRNA gene was extracted and amplified (Figure 2b), which showed 99.5% homology with *L. plantarum* JCM 1148. A phylogenetic tree was constructed to confirm its taxonomic classification. Multiple sequence alignment was conducted with ClustalX 2.1 (ClustalW algorithm, default parameters), and the tree was built using the neighbor-joining (NJ) method in MEGA 11, with 1,000 bootstrap replicates and the Kimura 2-parameter model for genetic distance calculation (Figure 2c). Based on these findings, the strain was designated as *L. plantarum* ZZU-1. Then, the EPS produced by strain ZZU-1 named EPS-ZZU was extracted and then purified by Sepharose CL-6B column (2.5 × 60 cm). After extraction and purification, SEM and AFM were selected to characterize the EPS-ZZU. As shown in Figure 2d, EPS-ZZU exhibits a sheet-like morphology with rough, dense porous and irregular sponge-like surface. The AFM images presented similar results with SEM, which showed non-uniform and porous structures with average pore diameter of about 50 nm (Figure 2e), indicating that the molecules have aggregated and the multi-molecular structure is branched and entangled (Carracedo-Cosme and Pérez, 2024).

**FIGURE 3:**
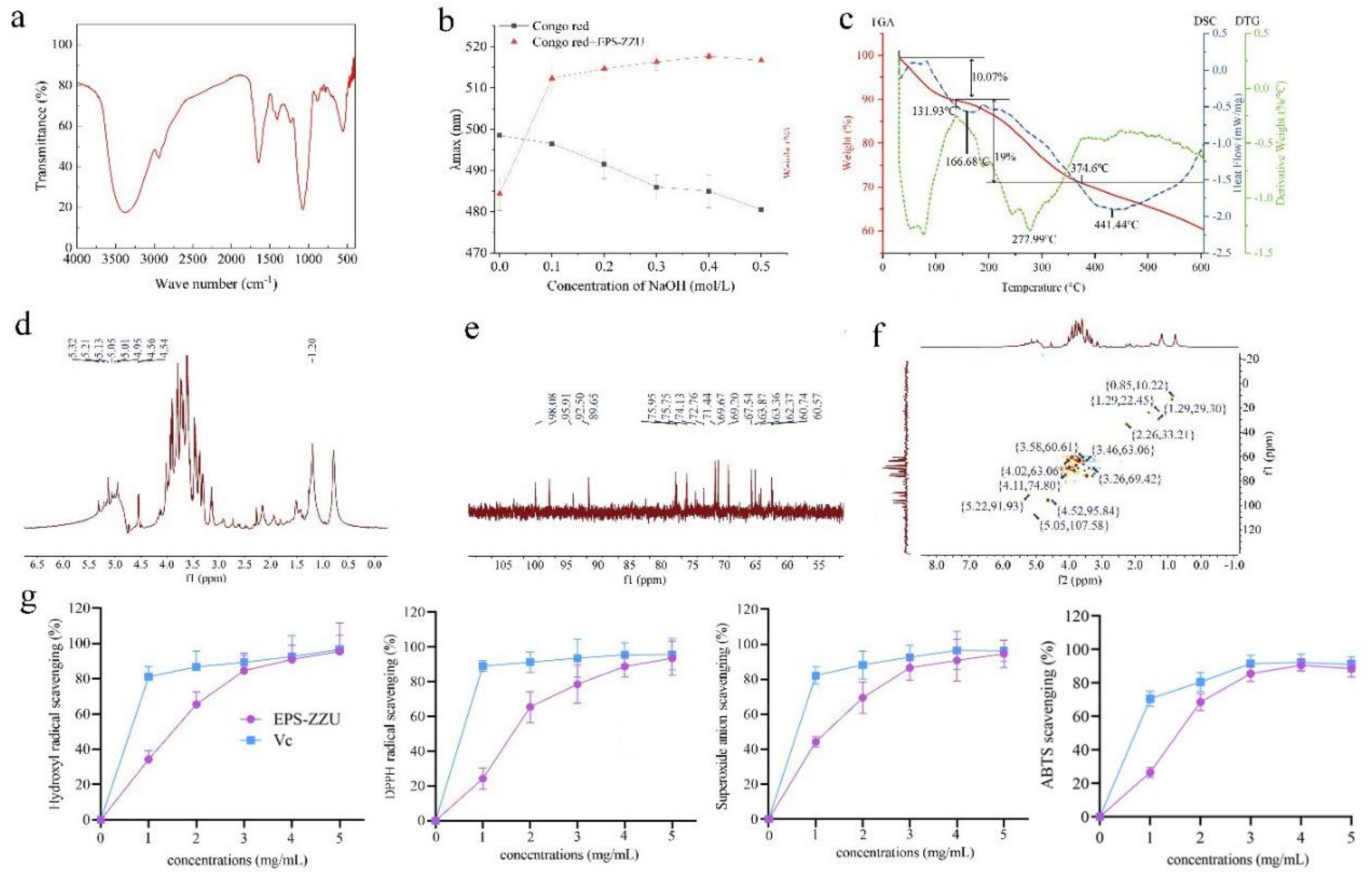
Characteristics of the EPS-ZZU. **(a)** FT-IR spectra, **(b)** triple helix conformation analysis, **(c)** ^1^H NMR, **(d)** ^13^C NMR, **(e)** HSQC, **(f)** thermal analysis, and **(g)** scavenging capacities against OH, DPPH•, O_2_^•⁣–^ and ABTS•^+^ free radicals of EPS-ZZU and vitamin C.

After purification, the characteristics of the EPS-ZZU was further analyzed. FTIR spectrum of EPS-ZZU displayed a strong and broad absorption peak at 3,600–3,200 cm^–1^, corresponding to the O-H stretching vibration of hydroxyl groups. A weak absorption band at 3,000–2,800 cm^–1^ was assigned to the C-H stretching and bending vibrations of aliphatic hydrocarbons. The characteristic peaks at 1,700–1600 cm^–1^ were attributed to the stretching vibration of C = O groups. Notably, the fingerprint region ranging from 1,200 to 1,000 cm^–1^ presented typical carbohydrate characteristic absorptions: the absorption bands within this region were mainly ascribed to the stretching vibrations of C-O-C and C-O in pyranose rings. The absorption at ∼840 cm^–1^ indicated the presence of α-configured glycosidic bonds (Figure 3a). The HPLC results showed that EPS-ZZU is a heteropolysaccharide containing mannose, glucose, ribose, fucose, glucuronic acid, arabinose, galactose, xylose, rhamnose and galacturonic acid with molar ratios of 34.4: 26.35: 12.24: 8.19: 5.57: 5.13: 3.35: 1.45: 0.63: 0.09 (Supplementary Figure 1). The molecular weight (Mw) was 2.141 kDa based on GPC analysis (Supplementary Figure 2). As shown in Figure 3b, the UV-Vis of EPS-Congo red complexes was shifted as the NaOH concentrations increased and the maximum absorption was 517 nm obtained at the concentrations of 0.4 mol/L NaOH. After that, it gradually decreased with the NaOH concentration increased, indicating that the exopolysaccharide has a triple-helix structure (Ma et al., 2025). Then, UV-visible spectrum of EPS-ZZU solution at 4 mg/mL (200–400 nm) showed no obvious absorption bands appeared at 260 nm and 280 nm, indicating that the obtained EPS-ZZU contained almost no nucleic acid and protein impurities (Supplementary Figure 3). The thermal behavior of EPS-ZZU was investigated by TGA-DSC analysis (Figure 3c), and the decomposition process could be divided into three distinct stages. The first stage, occurring from room temperature to approximately 131.93°C, showed a mass loss of 10.07%, which was attributed to the evaporation of free and bound water molecules, accompanied by a corresponding endothermic event in the DSC curve. The second stage, between 131.93°C and 374.6°C, represented the major thermal decomposition phase, where a significant mass loss of about 19% was observed, corresponding to the depolymerization of the polysaccharide backbone, cleavage of glycosidic bonds, and dehydration of sugar rings, with the maximum decomposition rate at 166.68°C as indicated by the DTG curve. The third stage, occurring from 374.6°C to 600°C, involved further slow mass loss, which was ascribed to the carbonization of residual organic components and the decomposition of more stable carbonaceous structures, leading to the formation of a small amount of ash residue as the TGA curve gradually stabilized above 441.44°C. Figure 3d denoted ^1^H NMR result with a signal at δ3.0–5.5 ppm, which is the typical characteristic of sugar, and EPS was found to have obvious heteromeric proton (H-1) chemical shift signals at δ5.32, 5.21, 5.13, 5.05, 5.01, 4.95, 4.56, and 4.54 ppm. The other signals at δ3.1–4.2 ppm represented the remaining protons, which were assigned to H-2-H-6. The peak at δ1.2 ppm was assigned as the proton signal of the methyl group in the fucose residue, and the δ4.8–5.5 ppm was a α-configuration pyran unit. In the ^13^C spectrum of EPS-ZZU (Figure 3e), the typical signal of sugar appears at δ60–100 ppm; and EPS-ZZU has four obvious chemical shift signals of heteropolymeric carbon signals (C-1) at δ98.08, 95.91, 92.5, and 89.65 ppm. As showed in the HSQC spectrum (Figure 3f), the anomeric carbon signals at δ107.58, 95.84, and 91.93 ppm are cross-linked with the anomeric proton signals at δ5.05, 5.52, and 5.22, respectively. To evaluate the antioxidant capacity of EPS-ZZU, its free radical scavenging activities against ⋅OH, DPPH•, O_2_^•⁣–^, and ABTS•^+^ were assessed and compared with Vc. The results demonstrated that EPS-ZZU achieved over 90% removal rates for all three free radicals when the concentrations was increased to 5 mg/mL (Figure 3g), which were comparable to those of Vc (5 mg/mL)without significant differences. These findings indicate that EPS-ZZU in 5 mg/mL concentrations possesses robust antioxidant properties, making it a promising antioxidant candidate in ASD intervention.

The structural characterization, conformation, morphology, and antioxidant potential of EPS-ZZU were systematically analyzed. In terms of structural characterization, EPS-ZZU is identified as a heteropolysaccharide composed of 10 monosaccharides, with mannose and glucose as the dominant components (molar ratios 34.4 and 26.35, respectively), consistent with the common structural feature of LAB-derived EPS that are mostly heteropolysaccharides dominated by glucose and mannose (Wang et al., 2023), but unlike most *L. plantarum* EPS (e.g., those from co-cultured *L. plantarum* CD11 and MT35) which mainly consist of glucose and mannose with fewer monosaccharide types (Xiong et al., 2023). EPS-ZZU contains additional rare monosaccharides including ribose, fucose and glucuronic acid, showing higher structural diversity, and its molecular weight (Mw) of 2.141 kDa is smaller than that of most *L. plantarum*-derived EPS (e.g., 14.639 kDa for EPS from *L. plantarum* HZB-30 and 84.2 kDa for EPS from *L. helveticus* WXD191), suggesting potential advantages in solubility and bioavailability (Xiao et al., 2022). Moreover, the Congo red test confirmed that EPS-ZZU has a triple-helix structure, a crucial structural feature for the biological activity of LAB-derived EPS (consistent with most reported *L. plantarum* EPS such as those from *L. plantarum* C88 which possess triple-helix or α-helix conformations), but the maximum absorption wavelength of EPS-ZZU-Congo red complex at 0.4 mol/L NaOH is 517 nm, slightly different from that of other *L. plantarum* EPS, indicating subtle differences in the spatial configuration of the triple-helix structure possibly due to its unique monosaccharide composition and glycosidic linkage mode (Guo et al., 2021). FTIR and NMR results further confirmed its typical sugar functional groups and α-configuration pyran units (consistent with *L. plantarum* and other LAB EPS) while its distinct chemical shift signals of heteromeric protons and carbons reflect unique structural arrangement; regarding morphology, although direct observation of EPS-ZZU’s microstructure was not conducted, its small Mw and high monosaccharide diversity suggest a relatively loose and flexible morphological structure, different from the compact and smooth surface structure of some *L. plantarum* EPS that affect the viscosity of fermented products (most *L. plantarum*-derived EPS exhibit rough and porous or smooth surface morphologies whose spatial structure affects functional properties like viscosity and water-holding capacity) (Yao et al., 2021). For antioxidant potential, EPS-ZZU achieves over 90% scavenging rates for O_2_^–⁣⋅^, ⋅OH, and DPPH free radicals at 5 mg/mL, comparable to Vc and superior to most reported EPS from *L. plantarum* and other LAB (e.g., EPS from *L. plantarum* EA3 shows strong radical scavenging ability but not reaching EPS-ZZU’s level, and most LAB-derived EPS require higher concentrations for similar effects), with its excellent antioxidant activity possibly related to its triple-helix conformation and rich hydroxyl groups that effectively chelate free radicals and reduce oxidative damage, collectively indicating that EPS-ZZU has unique structural characteristics and outstanding antioxidant capacity compared with EPS from *L. plantarum* and other LAB, making it a more promising candidate for antioxidant application (Villaño et al., 2007).

### 3.2 EPS-ZZU administration improved autism-like behavior in ASD rats

**FIGURE 4:**
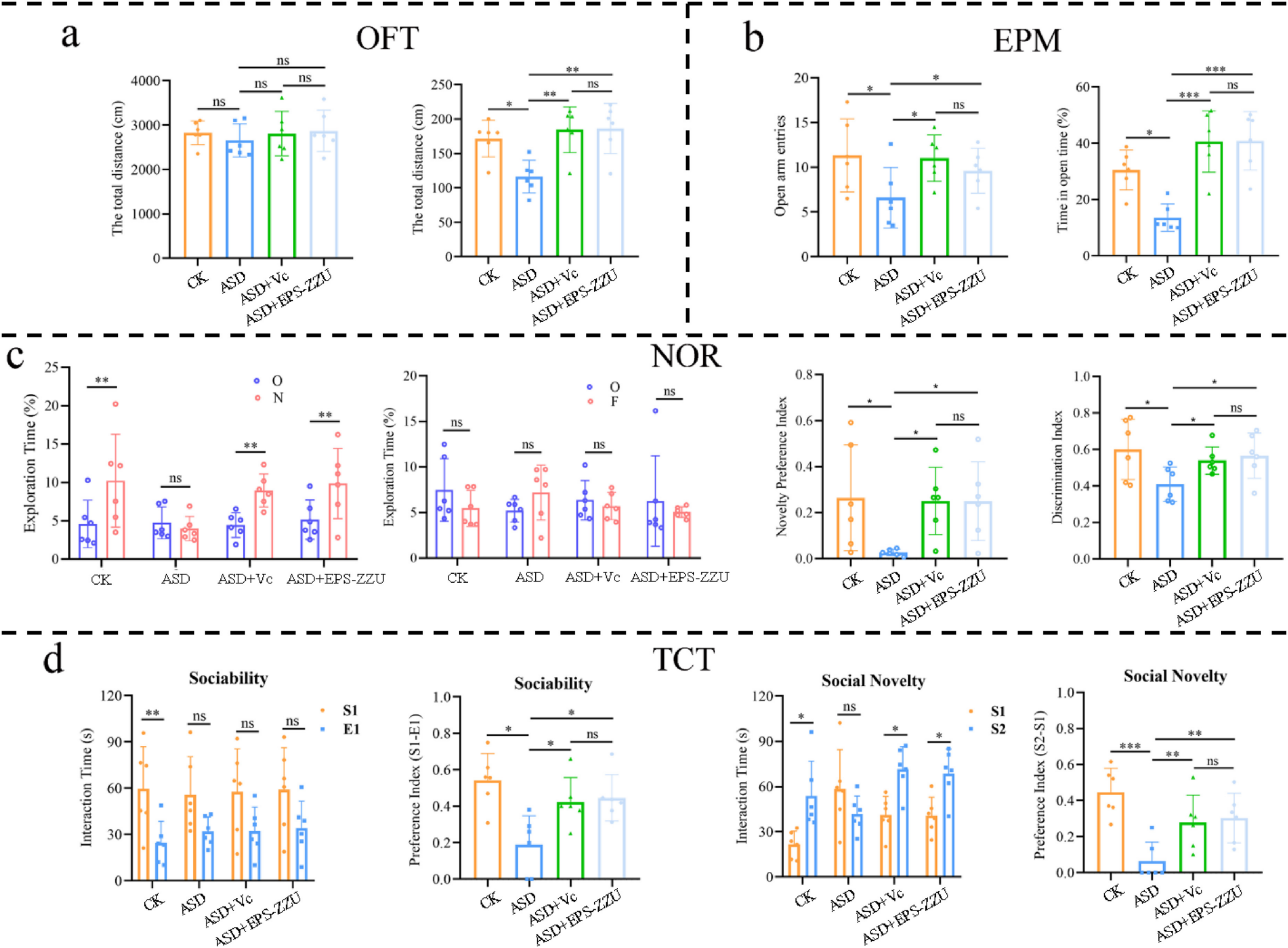
Behavioral tests in rats across different groups. **(a)** Results of the open field test (OFT), **(b)** results of the elevated plus maze test (EPM) test, **(c)** results of the novel object recognition (NOR) test, **(d)** results of the three-chamber social test (TCT). Multiple pairwise comparisons were performed using one-way ANOVA followed by Tukey’s *post-hoc* test. *p < 0.05, **p < 0.01, ***p < 0.001, ns, not significant.

The therapeutic efficacy of EPS-ZZU in VPA-induced ASD rats was evaluated using a comprehensive battery of behavioral assessments, including the OFT, EPM, NOR, and TCT. As illustrated in Figure 4a, the VPA-treated group exhibited a significant reduction in total locomotor activity (*P* < 0.05) compared to CK rats, while EPS-ZZU administration reversed this deficit by increasing total traveled distance (*P* < 0.05). In the EPM, the ASD group showed decreased entries and dwell time in the open arms, indicative of heightened anxiety and impaired exploratory behavior. However, EPS-ZZU treatment significantly enhanced these parameters, suggesting amelioration of anxiety-related deficits (Figure 4b). The NOR test revealed that VPA-induced rats displayed poor novelty recognition, as evidenced by reduced discrimination index between familiar and novel objects. EPS-ZZU intervention significantly improved this deficit, indicating enhanced cognitive flexibility (Figure 4c). Regarding social behavior, the TCT results demonstrated that VPA-treated rats exhibited reduced social interaction and social novelty preference, characterized by shorter latency to investigate unfamiliar rats and fewer interactions with novel conspecifics. In contrast, EPS-ZZU administration significantly restored these deficits, with increased social engagement and stronger social novelty discrimination (*P* < 0.05) (Figure 4d). The behavioral improvements observed in the EPS-ZZU treated group suggest effectively mitigates the core symptoms of ASD, including social deficits and repetitive behaviors.

Autism spectrum disorder is a neurodevelopmental disorder characterized by enduring impairments in social communication, restricted interests, and repetitive behaviors, with its pathogenesis involving intricate interactions between genetic predispositions and environmental influences (Simmons et al., 2021). The validation of behavioral assessment tools, including the OFT, EPM, NOR, and TCT, has established standardized methodologies for evaluating ASD-related phenotypes in rodent models (Feng et al., 2022). In this study, the EPS-ZZU was evaluated for its therapeutic potential in VPA-induced ASD rats. The results demonstrated that EPS-ZZU significantly ameliorated social interaction impairments, as evidenced by restored sociability and social novelty-seeking behaviors in the three-chamber test. Additionally, EPS-ZZU treatment increased locomotor activity in the OFT, indicating enhanced motor coordination, while also reducing anxiety-like behaviors by increasing the open-arm entries frequency and time spent with EPM. Moreover, EPS-ZZU intervention further improved cognitive flexibility through enhanced NOR, suggesting a multifaceted impact on neurobehavioral phenotypes (Liu et al., 2024). Notably, the therapeutic effects of EPS-ZZU were comparable to those of Vc, a known antioxidant. These results underscores the potential of EPS-ZZU as an adjunct therapy for ASD.

### 3.3 EPS-ZZU mitigated oxidative stress and neuroinflammation of ASD rats

**FIGURE 5:**
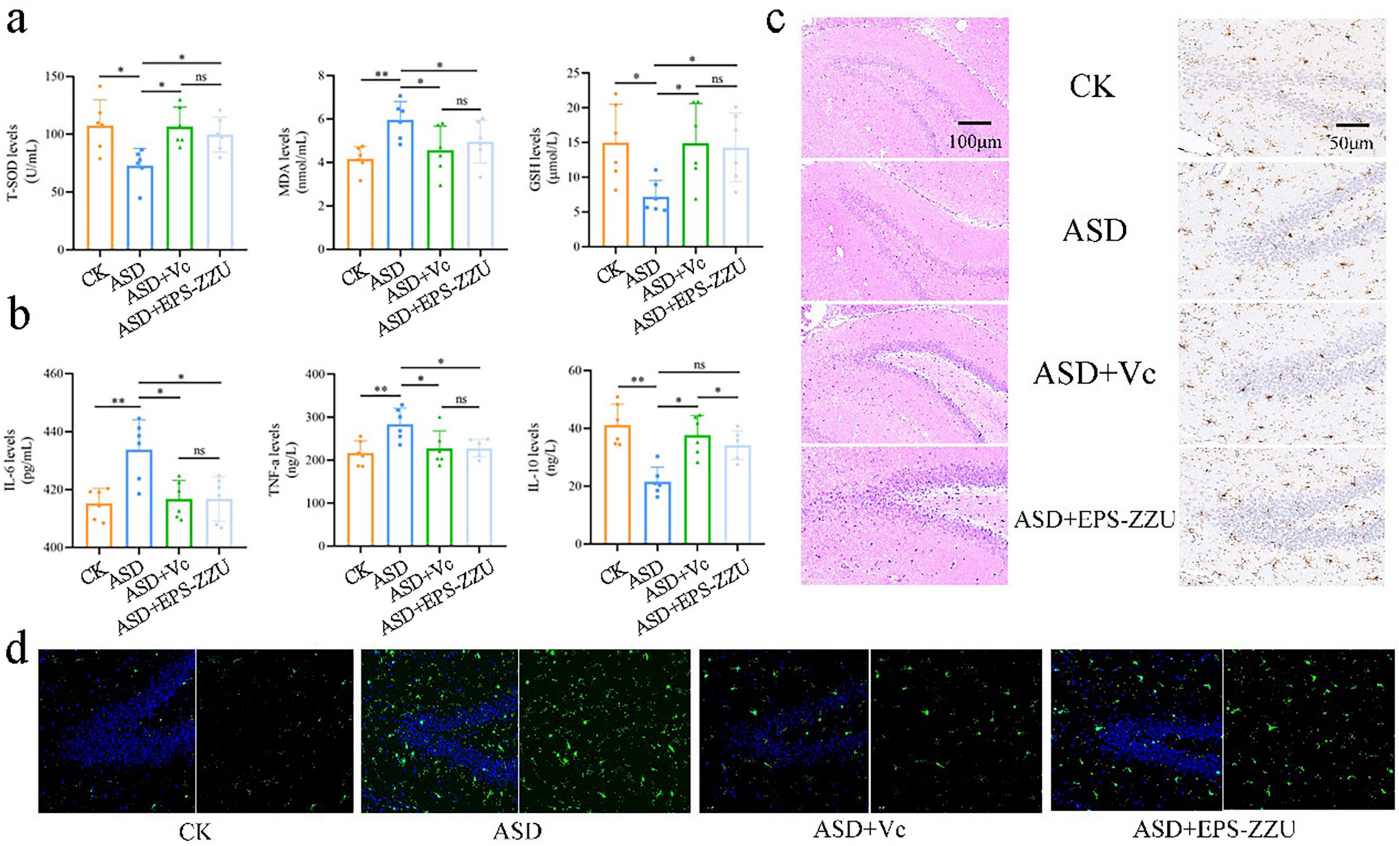
The EPS-ZZU of strain ZZU-1 effectively alleviated oxidative stress and neuroinflammation in autism spectrum disorders (ASD) rats. The levels of total superoxide dismutase (T-SOD), malondialdehyde (MDA), glutathione (GSH) **(a)** as well as interleukin-6 (IL-6), tumor necrosis factor-alpha (TNF-α), interleukin-10 (IL-10) **(b)**. Brain tissue sections were subjected to hematoxylin and eosin (H&E) staining **(c)**, immunohistochemical staining, and immunofluorescence assays **(d)** for histopathological evaluation. Multiple pairwise comparisons were performed using one-way ANOVA followed by Tukey’s *post-hoc* test. *p < 0.05, **p < 0.01, ns, not significant.

To further evaluate the EPS-ZZU for its effect in mitigating oxidative stress and attenuating neuroinflammation, key oxidative stress biomarkers including GSH, SOD, and MDA were assessed alongside inflammatory factors like TNF-α, IL-6, and IL-10. VPA administration significantly elevated oxidative stress markers (*P* = 0.0042, 0.0063, and 0.015 for GSH, T-SOD, and MDA, respectively) and pro-inflammatory cytokines (*P* = 0.0119, 0.0236, and 0.0303 for IL-6, IL-10, and TNF-α), indicating a robust inflammatory response. In contrast, EPS supplementation significantly restored GSH levels (14.37 ± 0.43 vs. 6.96 ± 0.61 μmol/L, *P* = 0.0201), decreased MDA (4.11 ± 0.11 vs. 6.03 ± 0.21 μmol/L, *P* = 0.0151), and enhanced SOD activity (114.55 ± 2.59 vs. 56.61 ± 4.42 U/mL, *P* = 0.0306) compared to the ASD group (Figure 5a). Similarly, EPS markedly reduced TNF-α, IL-6, and IL-10 levels, suggesting its anti-inflammatory potential. The levels of IL-6, IL-10, and TNF-α were significantly reduced after 1 month of treatment with EPS (*P* = 0.0119, 0.0236, and 0.0303, respectively) compared to the ASD group findings (Figure 5b). Histopathological examination of the hippocampus demonstrated that VPA-induced ASD resulted in disorganized and necrotic neurons within the dentate gyrus (DG) region. In contrast, EPS-ZZU treatment mitigated neuronal atrophy, as indicated by a more compact arrangement of neurons (Figure 5c). Concordantly, microglial density in the hippocampal DG region was significantly elevated in ASD model rats, yet markedly reduced following probiotic intervention (Figure 5d). Notably, the antioxidant and anti-inflammatory effects of EPS-ZZU were comparable to those of Vc, with no significant differences observed across most indices. These results highlights the feasibility of EPS-ZZU for targeted oxidative stress and therapeutic interventions in neurodevelopmental disorders.

Previous research has demonstrated that prenatal exposure to VPA induces ASD through oxidative stress and inflammatory response (Mirza and Sharma, 2019). Specifically, ASD are characterized by neuronal damage due to oxidative stress (Liu et al., 2023). Oxidative stress was noted to modify the inflammatory response. Even though oxidative stress and neuroinflammation are two totally different pathological events, they are linked and affect one another (Teleanu et al., 2022). Neuroinflammation together with oxidative stress are fundamental aspects that need to be taken into consideration regarding the onset and progression of neurodegenerative disorders, being inextricably linked in their pathogenesis (Solleiro-Villavicencio and Rivas-Arancibia, 2018). Reactive species secreted by inflammatory cells are key inducers of oxidative stress. Notably, specific ROS can amplify intracellular signaling cascades, thereby driving the upregulation of pro-inflammatory gene expression (Saxena et al., 2022). Thus, neuroinflammation and oxidative stress can stimulate one another, especially in the diseased state. The oxidative stress biomarkers (GSH, SOD, MDA) and inflammatory markers (TNF-α, IL-6, IL-10) form a coordinated network to reflect the crosstalk between oxidative stress and neuroinflammation: SOD, as a key antioxidant enzyme, scavenges excessive ROS to prevent oxidative damage, while GSH functions as a major intracellular antioxidant to maintain redox homeostasis, and their downregulation directly indicates impaired antioxidant capacity and enhanced oxidative stress (Demirci-Çekiç et al., 2022). MDA, a product of lipid peroxidation, serves as a reliable marker of oxidative damage to neuronal cell membranes, reflecting the severity of oxidative stress-induced neuronal injury (Tsikas, 2017). On the other hand, the pro-inflammatory cytokines TNF-α and IL-6 are key mediators of neuroinflammation, whose upregulation indicates the activation of inflammatory responses in the brain, and they can further promote the production of ROS by activating inflammatory cells, thereby exacerbating oxidative stress (Ge et al., 2018). IL-10, an anti-inflammatory cytokine, counteracts the pro-inflammatory effects of TNF-α and IL-6, and its expression level reflects the compensatory regulatory mechanism of the inflammatory system to limit excessive inflammation, which in turn reduces ROS production and alleviates oxidative stress (York et al., 2024). Collectively, these biomarkers complement each other mechanistically: oxidative stress biomarkers (GSH, SOD, MDA) directly reflect the degree of redox dysregulation and neuronal damage, while inflammatory markers (TNF-α, IL-6, IL-10) reveal the status of neuroinflammation that interacts with oxidative stress, together providing a comprehensive insight into the pathological processes of VPA-induced neurotoxicity in ASD model rats. Given that oxidative stress induction is likely a critical mechanism underlying VPA-induced neurotoxicity in ASD model rats, therapeutic strategies employing antioxidants, such as vitamin A, to mitigate oxidative stress have garnered increasing interest (Cheng et al., 2021). In the present study, the EPS exhibiting high antioxidant capacity, produced effects comparable to that of Vc-treated group. These findings underscore the critical role of redox dysregulation in VPA-induced neurotoxicity and highlight the efficiency of EPS-ZZU approaches targeting oxidative stress in ASD management.

### 3.4 EPS-ZZU protected intestinal function of ASD rats

**FIGURE 6:**
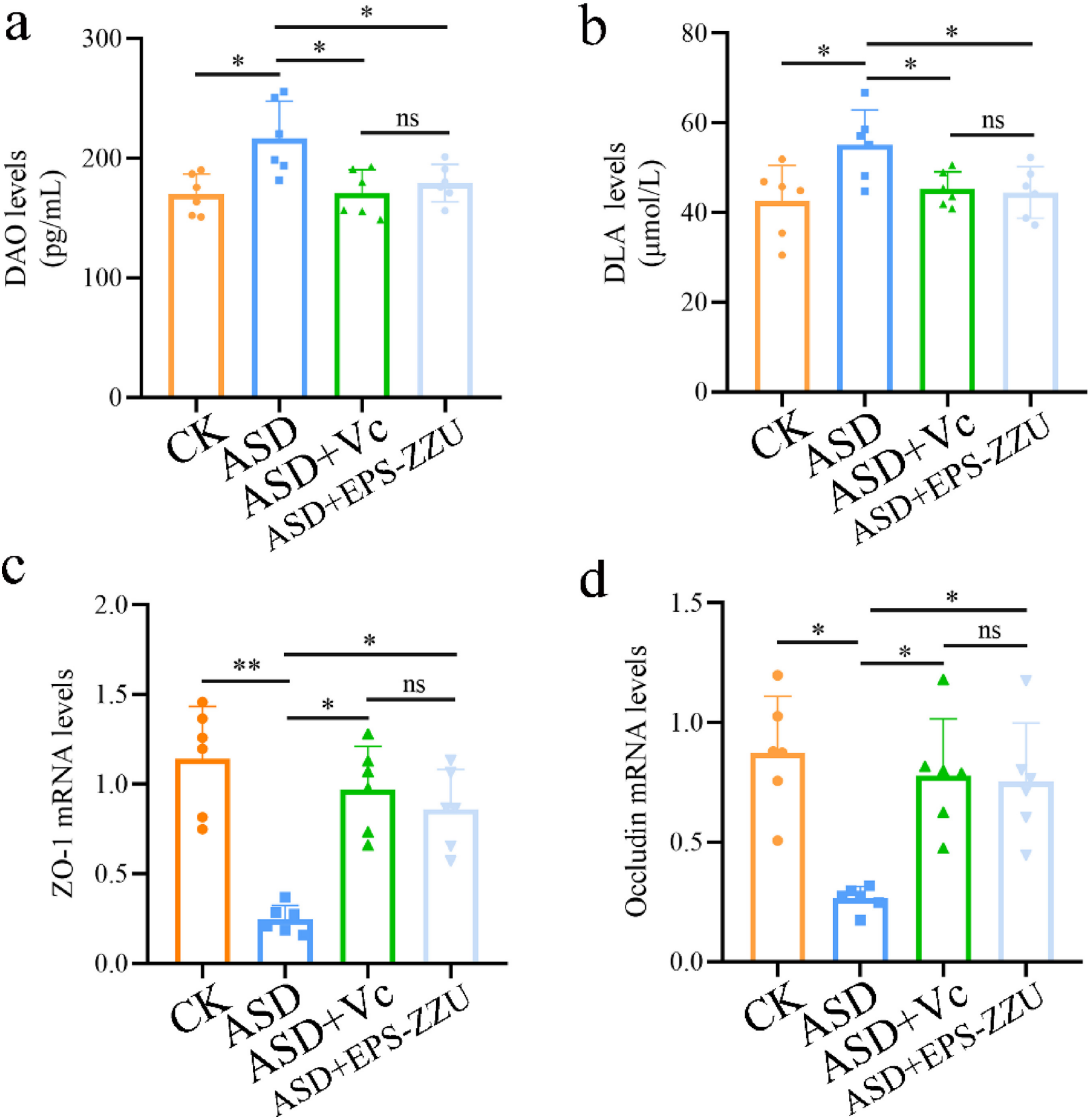
Intestinal permeability analysis of rats in different groups. Levels of diamine oxidase (DAO) **(a)**, DLA **(b)**, mRNA expression of ZO-1 **(c)**, and occludin **(d)** in the colon (*n* = 3 per group); Multiple pairwise comparisons were performed using one-way ANOVA followed by Tukey’s *post-hoc* test. *p < 0.05, **p < 0.01, ns, not significant.

To evaluate gut barrier function in various groups, the integrity of the intestinal barrier was quantified with DAO and DLA. As shown in Figures 6a, b, VPA-induced ASD resulted in significantly elevated DAO levels (223.370 ± 18.81 pg/mL, *P* = 0.0396) and DLA concentrations (57.79 ± 4.11 μmol/L, *P* = 0.0116), indicating compromised gut barrier integrity. In contrast, the EPS-ZZU treatment reversed this trend, with DAO and DLA levels significantly reduced to 172.63 ± 22.36 pg/mL (*P* = 0.0462) and 42.51 ± 8.06 μmol/L (*P* = 0.0446), respectively. The expression of tight junction proteins ZO-1 and occludin, critical for maintaining intestinal barrier function, was further analyzed. As depicted in Figures 6c, d and both ZO-1 and occludin were downregulated in ASD rats (0.180 ± 0.052, *P* = 0.0235; 0.284 ± 0.0262, *P* = 0.0451), whereas their levels were significantly increased in the EPS-ZZU treated group (0.811 ± 0.113, *P* = 0.0369; 0.773 ± 0.0927, *P* = 0.0210). Of note, the EPS-ZZU group exhibited outcomes that were on par with those of the Vc treatment group, indicating a similar efficacy between the two interventions. Collectively, these results demonstrate that EPS-ZZU improves gut barrier function, thereby mitigating central nervous system damage associated with ASD.

The integrity of the gut barrier is critical for maintaining intestinal homeostasis and preventing the translocation of harmful substances into systemic circulation, which has profound implications for neurological function (Seethaler et al., 2021). Biomarkers such as DAO and DLA serve as key indicators of gut permeability, with elevated levels typically reflecting compromised barrier function (Nagpal and Yadav, 2017). Treatment with EPS-ZZU demonstrated a restorative effect on gut barrier function, as evidenced by the significant reduction in DAO and DLA levels, indicating improved permeability and enhanced barrier integrity (Chelakkot et al., 2018). This improvement was further corroborated by the upregulated expression of tight junction proteins ZO-1 and occludin-key components of the intestinal barrier that modulate paracellular transport and preserve epithelial integrity (Kuo et al., 2022). Previous study reported that EPS with diverse composition and chain length isolated from various bacterial sources has been studied for its diverse bioactivities by promoting gut barrier dysfunction and endotoxin induced inflammation. It maintains a functional gut barrier by mechanisms involving promoting gut commensal populations, reducing pathobiont abundance, and lessening mucosal oxidative and inflammatory injury (Dey, 2020). The properly functioning barrier prevents the entry of toxins and pathogens into the bloodstream, thereby mitigating immune responses and neuroinflammation-key factors implicated in ASD pathogenesis. By improving gut barrier integrity, EPS may not only sustain intestinal health but also provide neuroprotection via the gut-brain axis (Banker et al., 2021). These findings indicate that EPS-ZZU may represent a promising therapeutic approach for ASD, through restoring intestinal homeostasis and function.

### 3.5 EPS-ZZU modulated VPA-induced gut dysbiosis in ASD rats

**FIGURE 7:**
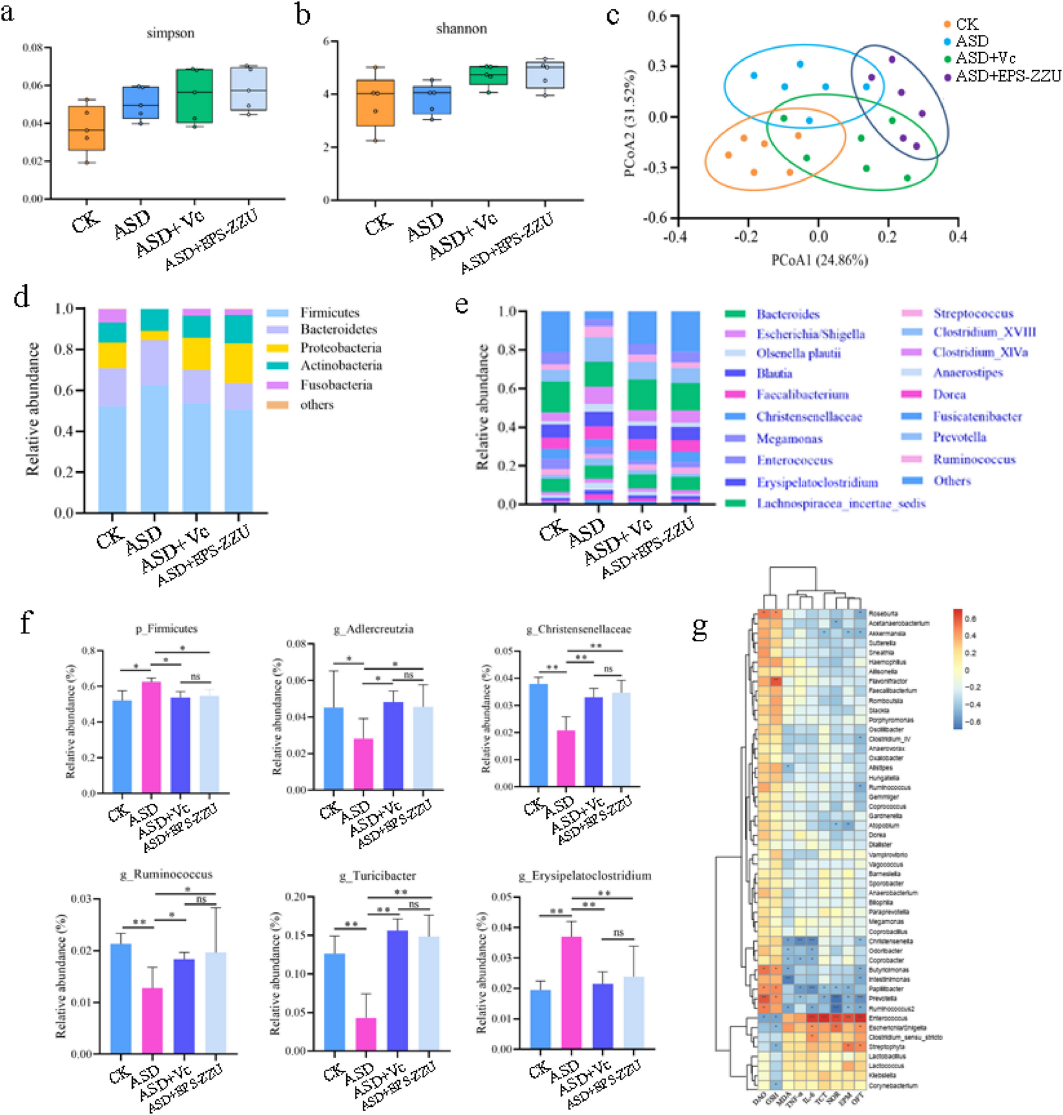
EPS-ZZU ameliorated gut microbiota dysbiosis in autism spectrum disorders (ASD) rats after intervention. The a-diversity of gut microbiota based on Simpson **(a)** and Shannon **(b)** indices. **(c)** The b-diversity of gut microbiota based on PCoA. Relative abundance of the intestinal microbial community in the each groups at the phylum **(d)** and genus **(e)** level. **(f)** Bacteria with differential abundance in the each groups. Data are shown as mean ± SD. **(g)** Heatmap of correlation between gut microbiota and several parameters like behave test and biochemical index in EPS-ZZU group. Multiple pairwise comparisons were performed using one-way ANOVA followed by Tukey’s *post-hoc* test. *p < 0.05, **p < 0.01, ns, not significant.

To evaluate the impact of EPS-ZZU on gut microbiota and its association with ASD-like behaviors, fecal samples were subjected to 16S rRNA gene sequencing to characterize microbial diversity and composition. The analysis revealed 1662 OTUs across all groups. Simpson and Shannon diversity indices demonstrated significantly higher alpha-diversity in the EPS-ZZU intervention group compared to the ASD group, indicating enhanced microbial richness and evenness (Figures 7a, b). β-diversity analysis via PCoA further confirmed distinct clustering patterns between the ASD and EPS-ZZU groups, with an Adonis test showing significant separation (*P* = 0.044), suggesting substantial alterations in gut microbial community structure following EPS-ZZU treatment (Figure 7c). At the phylum level, the relative abundances of *Bacteroidetes, Firmicutes*, and *Proteobacteria* were quantitatively assessed, revealing characteristic shifts in these dominant phyla between the experimental groups (Figure 7d). Compared to CK group, the relative abundance of *Firmicutes* in the ASD group exhibited a significant increase from 55.63% to 61.52% (Figure 7f). The *Firmicutes/Bacteroidetes* ratio was 3.28 in ASD group. Following treatment, this ratio was partially restored that was 2.81 in EPS-ZZU group (Supplementary Figure 4), suggesting a normalization of gut microbial homeostasis. At the genus level, the EPS-ZZU treated group displayed a distinct microbial community structure compared to the ASD group (Figure 7e). The results demonstrated that pathogenic bacteria was elevated and reduced beneficial species in ASD rats. Conversely, EPS-ZZU reversed the declines of beneficial bacteria such as *Adlercreutzia* (0.043% ± 0.005, *P* = 0.036), *Christensenellaceae uncultured* (0.034% ± 0.003, *P* = 0.023), *Turicibacter* (0.144% ± 0.017, *P* = 0.019), and *Ruminococcus* (0.017 ± 0.003, *P* = 0.041). Additionally, the relative abundance of harmful bacteria like *Erysipelatoclostridium* (0.022 ± 0.005, *P* = 0.016) was significantly reduced in the EPS-ZZU treated group. The EPS-ZZU intervention significantly modulated the gut microbiota composition, leading to a notable increase in beneficial bacterial taxa such as *Adlercreutzia, Christensenellaceae, Ruminococcus*, and *Turicibacter*. These microbial shifts were associated with a reduction in oxidative stress markers and inflammatory mediators, as evidenced by correlation analyses between gut microbiota and biochemical parameters (Figure 7g). Specifically, *Adlercreutzia* demonstrated positive correlations with both antioxidant indices (GSH and MDA) and behavioral assessments (TCT, NOR, EPM, and OFT), suggesting its role in mitigating oxidative stress and modulating gut-brain axis communication. Similarly, *Christensenellaceae, Ruminococcus*, and *Turicibacter* exhibited comparable trends in their association with reduced inflammation and improved behavioral outcomes.

Accumulating evidence supports the role of gut microbial dysbiosis in the pathogenesis of ASD (Morais et al., 2021). The bidirectional communication between the gut and the central nervous system, termed the “gut-brain axis” is increasingly recognized as a key pathway in ASD pathophysiology (Osama et al., 2025). The health beneficial effects of microbial EPS have been hypothesized to be associated with changes in gut microbial composition. Although most EPS are not easily digested or absorbed by saliva or the gastrointestinal tract, they act as prebiotics effectively utilized by gut microbiota to modulate the intestinal ecosystem and boost gut health (Tian et al., 2023). The mechanism by which EPS support a healthy gut micro-ecology involves indirect regulation of gut microbiota: they promote the growth and activity of beneficial microbes, which in turn enhances the diversity and stability of the gut microbial community (Tian et al., 2021). Prior reported that gut microbes can use EPS to produce short-chain fatty acid and maintain the gut barrier by attenuating mucosal inflammation and oxidative damage (Korakli et al., 2002). Moreover, within the intestinal lumen, bacterial EPS may also promote gut bacterial cross-feeding: bacteria unable to degrade EPS use the fermentation products of EPS-degrading strains as substrates to support their growth (Kaur and Dey, 2023). As evidenced by PCoA analysis, EPS-ZZU not only restores microbial diversity but also reshapes the overall microbial structure, a critical step toward normalizing gut-brain axis communication (Zeng et al., 2025). At the phylum level, the ASD group exhibited an elevated *Firmicutes/Bacteroidetes* (F/B) ratio, consistent with previous reports in ASD patients and animal models (Zhang et al., 2018). This imbalance is related to increasing of intestinal permeability and systemic inflammation, which may exacerbate ASD-like behaviors (Mlynarska et al., 2025). Notably, the enrichment of beneficial taxa in the EPS-ZZU group, which are associated with reduced oxidative stress (Zhang Z. et al., 2025), metabolic health (Sun et al., 2024) and neuroprotective effects (Xie et al., 2022), potentially contributing to the amelioration of ASD-like behaviors (Everett et al., 2022). Conversely, the reduction in *Erysipelatoclostridium* abundance following EPS-ZZU treatment that is linked to increased intestinal inflammation and systemic inflammation (Chen et al., 2023). The strong correlations between microbiota taxa and redox balance suggests a potential pathway: by enhancing antioxidant defenses to protect neurons from oxidative damage (Davinelli et al., 2025). These findings provide novel insights into the potential of EPS-ZZU as a microbiota-targeted intervention for ASD.

### 3.6 EPS-ZZU rectifies imbalances of gut microbiota-derived metabolites

**FIGURE 8:**
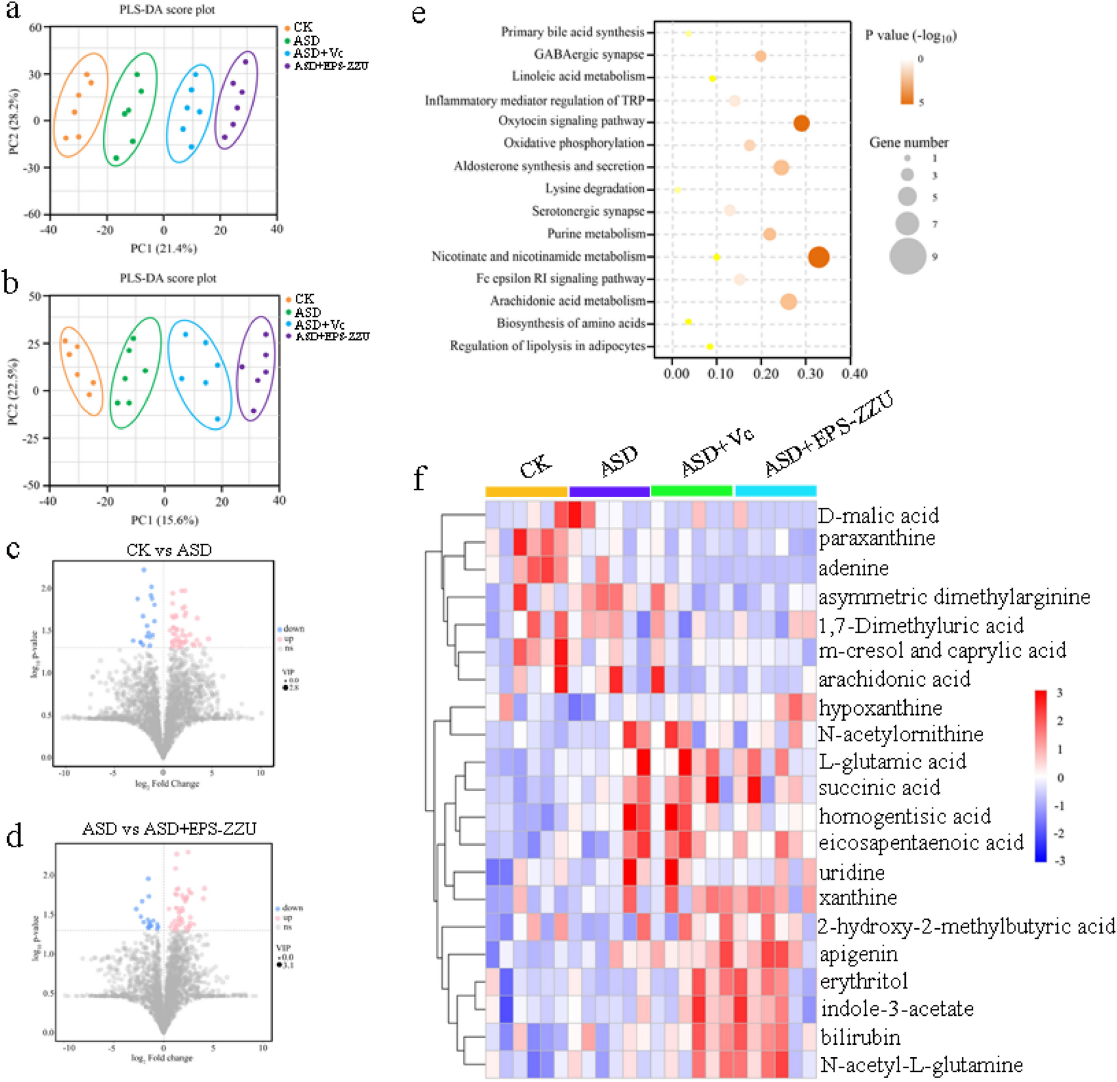
EPS-ZZU modulated metabolic processes in the gut microbiota of autism spectrum disorders (ASD) rats. PLS-DA clustering plots under negative **(a)** and positive **(b)** ion modes; Volcano plots illustrating fecal metabolome differences between the CK and ASD groups **(c)**, and between the ASD and EPS-ZZU groups **(d)**, red dots indicate significantly upregulated metabolites. **(e)** KEGG enrichment analysis of differential metabolites between the ASD and EPS-ZZU groups. **(f)** Differential metabolites across the four groups. Metabolites were considered differential with VIP > 1 and *P* < 0.05 (*t*-test).

To further investigate the effects of EPS-ZZU on gut microbiota, fecal metabolomic profiles were analyzed. As illustrated in Figures 8a, b PLS-DA score plots in both positive and negative ion modes demonstrated clear separation between the ASD and EPS-ZZU groups, indicating distinct metabolic profiles. A total of 83 differentially abundant metabolites was observed in ASD group, with 41 upregulated and 42 downregulated (Figure 8c), while 134 differential metabolites were detected after EPS-ZZU intervention, of which 81 were upregulated and 53 downregulated (Figure 8d). Functional enrichment analysis revealed 15 significantly enriched pathways, including primary bile acid biosynthesis, GABAergic synapse, and linoleic acid metabolism (Figure 8e). The impact of these metabolites on host physiology was further explored through correlation analysis with gut microbiota composition (Figure 8f). The results highlight the potential of EPS-ZZU in modulating gut-microbiota-metabolite interactions and function.

The current study demonstrate that EPS-ZZU intervention significantly reshapes the gut metabolic profile in ASD, with implications for key physiological pathways and potential therapeutic relevance. PLS-DA revealed a clear separation between the ASD and EPS-ZZU intervention groups in both positive and negative ion modes, indicating a distinct metabolic phenotype compared to the untreated ASD state. This separation suggests that metabolic perturbations associated with ASD are measurable and responsive to EPS-ZZU intervention, similar to previous studies demonstrating that gut microbiota-targeted therapies (e.g., probiotics) can modulate host metabolic profiles in neurodevelopmental disorders (Yadav et al., 2025). The greater number of differential metabolites detected in the EPS group further implies a broad regulatory effect on gut metabolism, potentially correcting pathological metabolic shifts while inducing novel beneficial adaptations. Metabolic pathway enrichment analysis identified 15 significantly enriched pathways, many of which are critically linked to ASD pathogenesis and gut-brain axis communication. Primary among these is the primary bile acid biosynthesis pathway, which plays a pivotal role in lipid metabolism, gut barrier integrity, and microbial balance (Jia et al., 2018). Dysregulated bile acid metabolism has been associated with ASD, as bile acids function as signaling molecules influencing neuroinflammation and neurotransmission (Jia and Xie, 2018). The enrichment of the GABAergic synaptic pathway is a notable finding, as GABA, a primary inhibitory neurotransmitter, plays a critical role in regulating neuronal excitability and is implicated in the pathogenesis of neurodevelopmental disorders like ASD (Giniatullin and Cherubini, 2025). EPS-ZZU enhances GABAergic neurotransmission, potentially alleviating neurobehavioral abnormalities. This aligns with evidence suggesting that gut microbiota, such as *Lactiplantibacillus* strains, can modulate GABAergic signaling and central nervous system function. Additionally, pathways related to linoleic acid metabolism and inflammatory mediator regulation of TRP channels highlight the anti-inflammatory of EPS-ZZU. Linoleic acid, an omega-6 fatty acid, serves as a precursor to anti-inflammatory lipids (e.g., resolvins), while TRP channels are critical for sensory processing and inflammation, both of which are disrupted in ASD (Rather et al., 2023). The observed alterations in metabolites, such as elevated indole-3-acetate and reduced levels of pro-inflammatory mediators (e.g., asymmetric dimethylarginine, homogentisic acid, and arachidonic acid), support the anti-inflammatory and neuroprotective of EPS (Simopoulos, 2002; Ueda et al., 2007). These findings suggest that EPS-ZZU modulates gut metabolism through interconnected pathways, influencing inflammation, neurodevelopment, and gut-brain axis function.

## 4 Conclusion

In this study, a novel exopolysaccharide named EPS-ZZU was obtained from *L. plantarum* ZZU-1, a probiotic strain isolated from traditional fermented “Suancai.” EPS-ZZU supplementation effectively ameliorates autism-like behaviors in mice by oxidative stress reduction and further decrease neuroinflammation through gut-brain axis. Moreover, EPS-ZZU also modulates gut microbiota composition by expanding populations of beneficial bacteria and diminishing harmful species. Concurrently, EPS-ZZU elevates levels of the beneficial metabolite indole-3-acetate, while reducing metabolites associated with cognitive impairment. The concentrations applied in the present work are within the physiologically safe and biomedically applicable range, which supports the potential application of EPS-ZZU in the prevention and improvement of cellular oxidative alterations. These findings underscore the potential of EPS-ZZU as a promising therapeutic agent for managing ASD via alleviating oxidative stress.

## Data Availability

16s RNA sequencing of strain ZZU-1 was deposited in NCBI under the accession number PV945894, https://www.ncbi.nlm.nih.gov/search/all/?term=PV945894.1. Raw gut microbiota sequencing data have been deposited in the NCBI Sequence Read Archive under accession number PRJNA1307800, http://www.ncbi.nlm.nih.gov/bioproject/1307800. The raw gut microbiota-related metabolomics data have been deposited in MetaboLights under the accession number MTBLS3662, https://www.ebi.ac.uk/ebisearch/search?db=allebi&sortignorenull=true&query=MTBLS3662.
