# Supplementary material for: Isolation and characterization of a novel exopolysaccharide from the fermented probiotic Lactiplantibacillus plantarum ZZU-1 and its application for attenuating autism-like behaviors
Source: Front Microbiol. 2026 Jul 8;17:1806688. doi: 10.3389/fmicb.2026.1806688 (PMC13388933; doi:10.3389/fmicb.2026.1806688)
Supplement: Supplementary file 1 [file Data_Sheet_1.docx]

Supplementary materials

Isolation and characterization of a novel exopolysaccharide from the fermented probiotic *Lactiplantibacillus plantarum* ZZU-1 and its application for attenuating Autism-like Behaviors


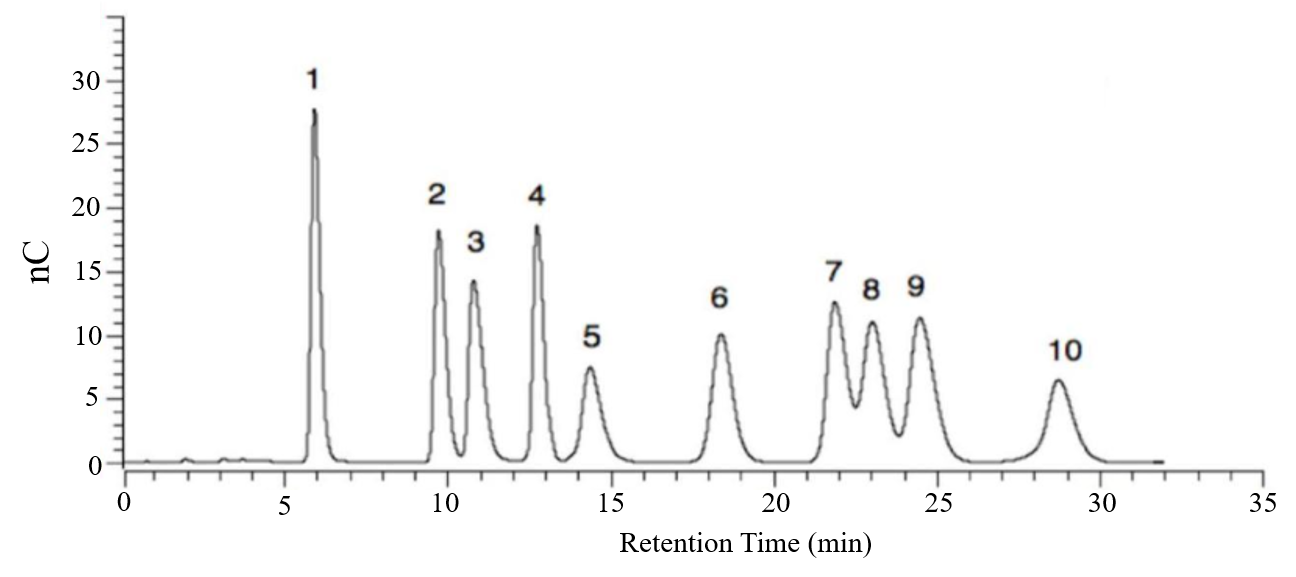


Fig.S1 HPLC chromatogram of monosaccharide composition of EPS-ZZU.


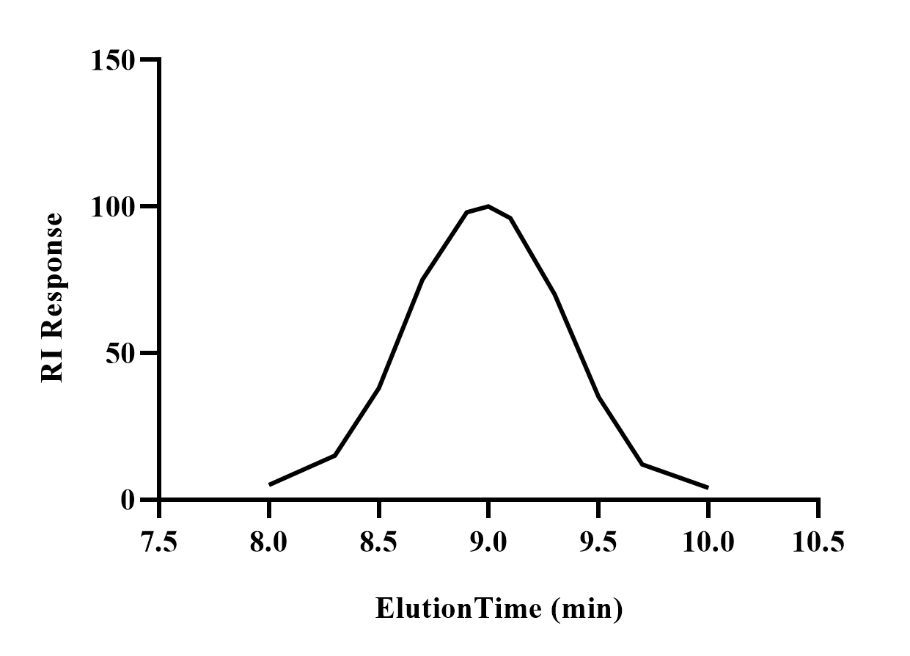


Fig.S2 HPSEC elution chromatogram for molecular weight determination of EPS-ZZU.


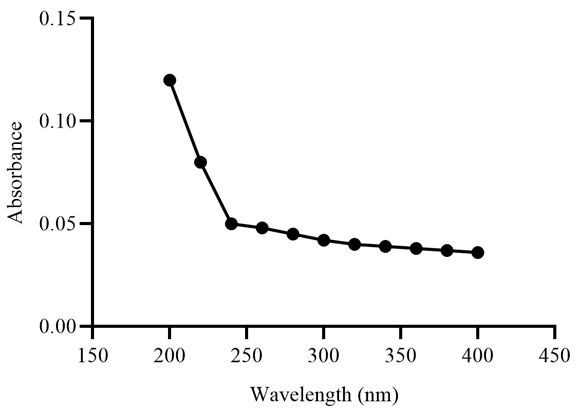


Fig.S3 UV-visible spectrum of EPS-ZZU solution (4 mg/mL) recorded from 200 to 400 nm.


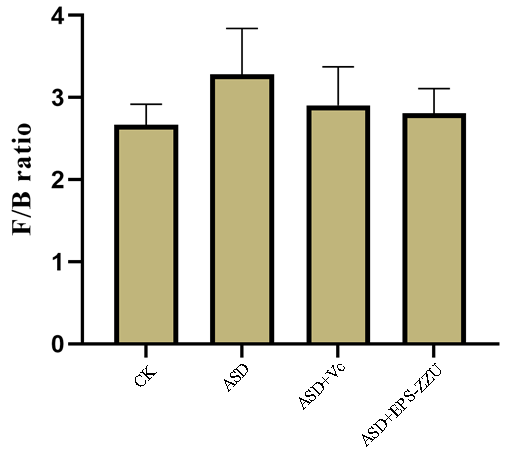


Fig.S4 The Firmicutes/Bacteroidetes ratio in different groups.
